# The hnRNP-Q Protein LIF2 Participates in the Plant Immune Response

**DOI:** 10.1371/journal.pone.0099343

**Published:** 2014-06-10

**Authors:** Clémentine Le Roux, Stefania Del Prete, Stéphanie Boutet-Mercey, François Perreau, Claudine Balagué, Dominique Roby, Mathilde Fagard, Valérie Gaudin

**Affiliations:** 1 INRA-AgroParisTech, UMR1318, Institut J.-P. Bourgin, Centre de Versailles-Grignon, Versailles, France; 2 INRA, Laboratoire des Interactions Plantes-Microorganismes (LIPM), UMR441, Castanet-Tolosan, France; 3 CNRS, Laboratoire des Interactions Plantes-Microorganismes (LIPM), UMR2594, Castanet-Tolosan, France; University of Georgia, United States of America

## Abstract

Eukaryotes have evolved complex defense pathways to combat invading pathogens. Here, we investigated the role of the *Arabidopsis thaliana* heterogeneous nuclear ribonucleoprotein (hnRNP-Q) LIF2 in the plant innate immune response. We show that *LIF2* loss-of-function in *A. thaliana* leads to changes in the basal expression of the salicylic acid (SA)- and jasmonic acid (JA)- dependent defense marker genes *PR1* and *PDF1.2*, respectively. Whereas the expression of genes involved in SA and JA biosynthesis and signaling was also affected in the *lif2-1* mutant, no change in SA and JA hormonal contents was detected. In addition, the composition of glucosinolates, a class of defense-related secondary metabolites, was altered in the *lif2-1* mutant in the absence of pathogen challenge. The *lif2-1* mutant exhibited reduced susceptibility to the hemi-biotrophic pathogen *Pseudomonas syringae* and the necrotrophic ascomycete *Botrytis cinerea*. Furthermore, the *lif2-1 sid2-2* double mutant was less susceptible than the wild type to *P. syringae* infection, suggesting that the *lif2* response to pathogens was independent of SA accumulation. Together, our data suggest that *lif2-1* exhibits a basal primed defense state, resulting from complex deregulation of gene expression, which leads to increased resistance to pathogens with various infection strategies. Therefore, LIF2 may function as a suppressor of cell-autonomous immunity. Similar to its human homolog, NSAP1/SYNCRIP, a *trans*-acting factor involved in both cellular processes and the viral life cycle, LIF2 may regulate the conflicting aspects of development and defense programs, suggesting that a conserved evolutionary trade-off between growth and defense pathways exists in eukaryotes.

## Introduction

Living organisms are exposed to various abiotic and biotic stresses and continuously integrate diverse signals to limit the spread of microbial pathogens. RNA regulatory processes, which modulate transcriptional programs and post-transcriptional events in response to various cues, are an important component of the tolerance and adaptation strategies of organisms. Thus, RNA-binding proteins (RBPs), which are involved in various aspects of RNA processing (i.e., mRNA maturation, editing, splicing, and mRNA trafficking), are thought to be key regulators of stress responses in both animals and plants. However, this possibility has been scarcely studied.

The heterogeneous nuclear ribonucleoprotein (hnRNP) group of RBPs forms a large family of ancestral modular proteins with a high degree of functional diversification [1]. In humans, this group contains 37 members, only a few of which are involved in the stress response. For instance, hnRNP-K participates in the response to genotoxic stress [2]. The human NS1-associated protein 1/synaptotagmin-binding cytoplasmic RNA-interacting protein (NSAP1/SYNCRIP), another cellular hnRNP protein, participates in the translational activation of both cellular and viral RNAs and functions in the Hepatitis C virus (HCV) life cycle by interacting with HCV RNA [3], [4], [5]. Knock-down of *NSAP1/SYNCRIP* significantly decreased the amount of HCV RNA in mammalian cells, suggesting that NSAP1/SYNCRIP is a negative regulator of viral defense responses. Recently, hnRNP-I was shown to interact with the long non-coding RNA *RoR* to modulate the expression of the tumor suppressor p53 in response to DNA damage and to participate in a surveillance network [6].

Plants contain more RBPs than do animals, but the number of plant hnRNPs is similar in both plants and animals. The functions of hnRNPs remain poorly described in plants [1], [7]. Few studies have reported a role for plant hnRNPs in the stress response [8]. Until now, the glycine-rich RBP AtGRP7 was the only hnRNP known to play a role in the plant's response to pathogens [9]. AtGRP7 is the substrate of the type III effector HopU1, which is injected by the bacterial pathogen *Pseudomonas syringae* into plant host cells. HopU1 mono-ADP ribosylates a conserved arginine residue of AtGRP7, preventing binding to immunity-related RNA [10], [11].

In a previous study, we identified LIF2, a novel hnRNP of the cruciferous plant *Arabidopsis thaliana* [12]. LIF2 is a nucleo-cytoplasmic RBP that contains three RNA Recognition Motifs (RRMs), the most frequent RNA-binding domain in the hnRNP family. LIF2 and its close homologs, AtLIL1 and AtLIL2, belong to the hnRNP-Q subfamily and LIF2 is structurally homologous to human NSAP1/SYNCRIP[12]. LIF2 interacts *in vivo* with LHP1, a Polycomb Repressive Complex1 (PRC1) subunit [13], [14]. LIF2 is involved in the maintenance of plant cell identity and cell fate decision [12]. Indeed, loss-of-function of *LIF2* affected various aspects of growth and development, such as flowering time and leaf size. More dramatically, *lif2* mutations induced indeterminate growth of ovaries, resulting in the formation of ectopic inflorescences bearing severely affected flowers. Besides its developmental functions, LIF2 might be involved in the stress response. Indeed, a large set of stress-related genes (193) was found to be deregulated in *lif2-1*. The set of common genes deregulated in both *lif2-1* and *lhp1* mutants was even more enriched in stress-related genes than was that in *lif2-1* [12]. Furthermore, loss-of-function of *LHP1* (also named *TFL2* or *TU8*) altered the glucosinolate profile and reduced symptoms in response to infection by the obligate biotrophic fungus *Plasmodiophora brassicae*, which causes clubroot disease, a damaging disease in Brassicaceae [15], [16].

These data suggest that LIF2 and LHP1 have common functions in the stress response and prompted us to investigate the function of LIF2 in biotic stress responses. Here, we show that *lif2* mutations conferred altered susceptibility to the necrotrophic fungal pathogens *Sclerotinia sclerotiorum* (*S. sclerotiorum*) and *Botrytis cinerea* (*B. cinerea*) and to the hemi-biotrophic bacterial pathogen *P. syringae* pv. *tomato* (*Pst*), three biological agents with substantial impacts on the agronomical production of various plant species [17], [18], [19]. To better understand the altered pathogen susceptibility of the *lif2* mutants, we investigated the expression of defense marker genes regulated by jasmonic acid (JA) and salicylic acid (SA), two critical signaling hormones in the activation of plant defense, as well as the production of stress-associated metabolites and hormones. Finally, we showed that the transcriptomic profiles of stress-response factors in *lif2* are specific and we identified key defense regulators (such as the *WRKY18* and *WRKY33* transcription factors, which are known to be involved in *B. cinerea* susceptibility), whose deregulation may contribute to the observed pathogen response. Together, our data suggest that the LIF2 hnRNP-Q may suppress the plant immune response by an unknown SA-independent pathway. Given that the human homolog of LIF2, the NSAP1/SYNCRIP protein, is a *trans*-acting factor involved in both cellular processes and the viral life cycle [3], [4], [5], we propose that the conserved hnRNP-Q proteins may have an evolutionary conserved function in regulating the trade-off between growth and defense in eukaryotes.

## Results

### Analysis of the *lif2-1* transcriptome reveals a potential stress-related function for LIF2

In a previous transcriptome profiling experiment, we showed that genes that were deregulated in the *A. thaliana lif2-1* null mutant were greatly enriched in Gene Ontology (GO) terms involved in responses to stress stimuli [12]. Here, we further examined the deregulated gene set (1008 genes) using Singular Enrichment Analysis (SEA) implemented in the agriGO toolkit [20]. This analysis revealed 293 deregulated genes associated with the GO term “response to stimulus” and 193 with “response to stress” (Table 1). We noticed that the normed frequency (NF) of the “response to biotic stimulus” (GO:000960, NF 4.22, p-value 7×10^−23^) was higher than that of the “response to abiotic stimulus” (GO:0009628, NF 2.75, p-value 2×10^−16^) (Table 1). Furthermore, several genes were associated with GO terms related to the JA-defense signaling pathway, the glucosinolate metabolic pathway, and responses to fungal and bacterial pathogens (Table 1). These data suggest that even if *LIF2* is induced only weakly upon pathogen infection (Fig. S1), this gene might be involved in defense responses to various bioaggressors.

**Table 1:** Gene ontology (GO) analysis of the deregulated genes in the *lif2-1* mutant.

Class	Term		Query item	Ref item	p-value	FDR	NF
Stimulus							
	Response to stimulus	GO:0050896	293	4057	8,60E-46	2,00E-42	2.78
	Response to chemical stimulus	GO:0042221	172	2085	9,20E-36	7.1e-33	3.17
	Response to biotic stimulus	GO:0009607	70	638	7,00E-23	2.3e-20	4.21
	Response to external stimulus	GO:0009605	52	429	2,10E-19	4.9e-17	4.66
	Response to endogenous stimulus	GO:0009719	87	1068	2,60E-19	5.4e-17	3.13
	Response to abiotic stimulus	GO:0009628	105	1471	1,00E-18	2,00E-16	2.75
Stress							
	Response to stress	GO:0006950	193	2320	5,00E-40	5.8e-37	3.20
	Response to wounding	GO:0009611	43	197	1,50E-26	7.1e-24	8.39
	Defense response	GO:0006952	72	766	1,10E-19	2.9e-17	3.61
Biotic stimulus							
	Response to fungus	GO:0009620	25	158	8,40E-13	1.2e-10	6.09
	Response to bacterium	GO:0009617	30	247	5,60E-12	7.2e-10	4.67
Hormones							
	Response to jasmonic acid stimulus	GO:0009753	33	215	5,80E-16	1,00E-13	5.90
	Response to hormone stimulus	GO:0009725	66	982	1,80E-11	2,00E-09	2.58
	Jasmonic acid mediated signaling pathway	GO:0009867	12	49	3,80E-09	2.9e-07	9.42
	Response to abscisic acid stimulus	GO:0009737	31	378	3,90E-08	2.5e-06	3,15
	Glycosinolate metabolic process	GO:0019757	12	62	6,50E-08	3.8e-06	7.44
Various							
	Response to chitin	GO:0010200	27	151	4,90E-15	8.1e-13	6.88
	Response to carbohydrate stimulus	GO:0009743	31	240	4,90E-13	7.6e-11	4.97
	Lipid localization	GO:0010876	11	24	7,10E-12	8.7e-10	17.63
	Response to water deprivation	GO:0009414	28	229	2,30E-11	2.5e-09	4.70
	Immune response	GO:0006955	36	367	2,60E-11	2.7e-09	3.77
	Response to osmotic stress	GO:0006970	38	408	3,60E-11	3.3e-09	3.58
	Response to salt stress	GO:0009651	33	366	1,40E-09	1.2e-07	3.47
	Response to oxidative stress	GO:0006979	31	332	1,90E-09	1.5e-07	3.59

Among the 1008 deregulated genes in *lif2-1*, 982 are associated with a GO term and were analysed using the agriGO toolkit. GO terms with the best p-value (p<10^−8^) were selected to illustrate *lif2* transcriptome specificities. Query item: number of deregulated genes in *lif2* in a given GO class. Reference item: total number of genes in a given GO class. The *A. thaliana* genome accounts for a total of 37767 items in the agriGO toolkit. The normed frequency (NF)  =  (Query item/Ref item)/(982/37767).

### JA and SA pathways are altered in the *lif2-1* mutant

**Figure 1:**
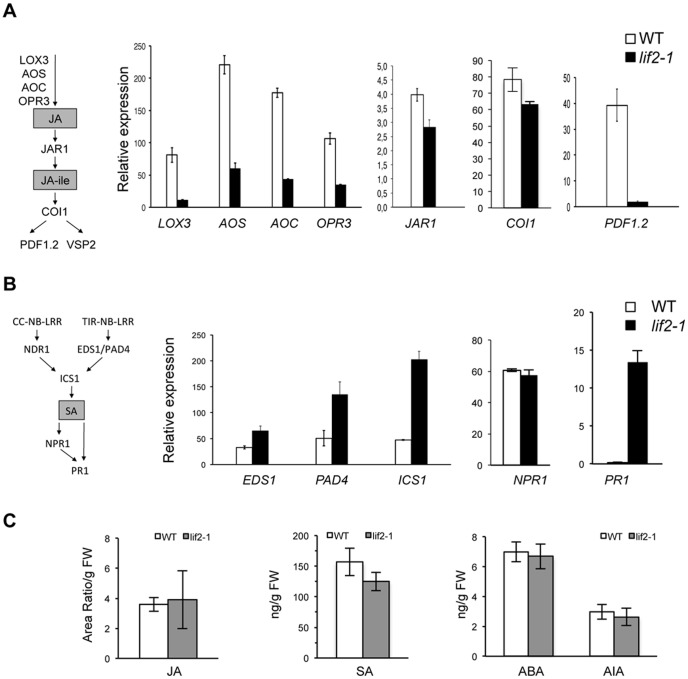
JA- and SA-dependent signaling pathways in the *lif2-1* mutant. (A) Expression levels of genes involved in the JA-dependent signaling pathway (*LOX3, AOS, AOC, OPR3, COI1*, and JAR1) and of a JA-responsive marker gene (*PDF1.2)* in the rosette leaves of seven-week-old wild-type (WT) and *lif2-1* plants. (B) Expression levels of genes of the SA-dependent signaling pathway (*EDS1*, *PAD4*, and *ICS1*) and of SA-responsive marker genes (*NPR1* and *PR1*) in seven-week-old wild-type (WT) and *lif2-1* rosette leaves. CC-NB-LRR and TIR-NB-LRR are disease resistance proteins with coiled-coil (CC), nucleotide-binding (NB), leucine-rich repeat (LRR), or Toll-Interleukin Receptor (TIR) domains. (C) Quantification of phytohormone contents in the rosette leaves of three-week-old plants using HPLC-electrospray-MS/MS. The amount of JA was expressed as a ratio of peak areas (209>62/214>62) per fresh weight (FW). The amount of other hormones was expressed in ng/FW. The bars represent standard deviation.

Based on the *lif2* transcriptome data [12], we further analyzed the expression of a key marker gene of the JA-mediated defense, *PDF1.2* (a JA-responsive gene), and the expression of genes of the JA biosynthesis and signaling pathways, *LOX3*, *AOS*, *AOC*, and *OPR3* (genes encoding enzymes of the JA biosynthesis pathway), *JAR1* (encoding an enzyme that converts JA to the bioactive JA-Ile molecule), and *COI1* (encoding an F-box subunit of the JA-receptor complex) [21] (Fig. 1A). All of these genes were downregulated in *lif2-1*, suggesting that the JA defense pathway is globally repressed in the *lif2-1* mutant.

Since the JA-dependent defense network cross-communicates with the SA-dependent signaling pathway to fine-tune the plant's defense response [22], [23], we assessed the expression of *ICS1/SID2*, which is involved in SA biosynthesis; *ENHANCED DISEASE SUSCEPTIBILITY1* (*EDS1*) and *PAD4*, which are involved in the SA-signaling pathway; and *NON-EXPRESSOR OF PR GENES 1* (*NPR1*) and *PATHOGENESIS-RELATED 1* (*PR1*), which are important positive regulators of SA responses [24], [25]. *EDS1*, *PAD4*, and *ICS1* were upregulated in the *lif2-1* mutant relative to wild-type plants (Fig. 1B). *PR1* was also upregulated in *lif2-1*, whereas *NPR1* expression was not significantly affected in *lif2-1* (Fig. 1B). These data suggest that most genes involved in the SA-mediated defense pathway are constitutively upregulated in the *lif2* mutants, in the absence of pathogen. The upregulation of genes of the SA signaling pathway and the downregulation of genes of the JA signaling pathway in *lif2* illustrates the antagonistic action of these two pathways.

Prompted by the finding that genes involved in JA- and SA-defense-related networks are differentially expressed in *lif2-1*, we quantified the endogenous levels of these hormones in *lif2-1* using HPLC-electrospray-MS/MS (Fig. 1C). Surprisingly, whereas *ICS1* was upregulated in *lif2-1*, free SA levels were similar in the mature rosette leaves of *lif2-1* and wild-type plants (Fig. 1C). Similarly, the JA levels were not significantly affected in the *lif2-1* mutant (Fig. 1C). Furthermore, we found that the levels of auxin (AIA) and abscissic acid (ABA), two other phytohormones recently shown to be involved in the stress response [26], [27], were not significantly altered in *lif2-1* in the absence of pathogen challenge (Fig. 1C).

### The *lif2* mutants are less susceptible to the bacterial pathogen *Pseudomonas syringae*

**Figure 2:**
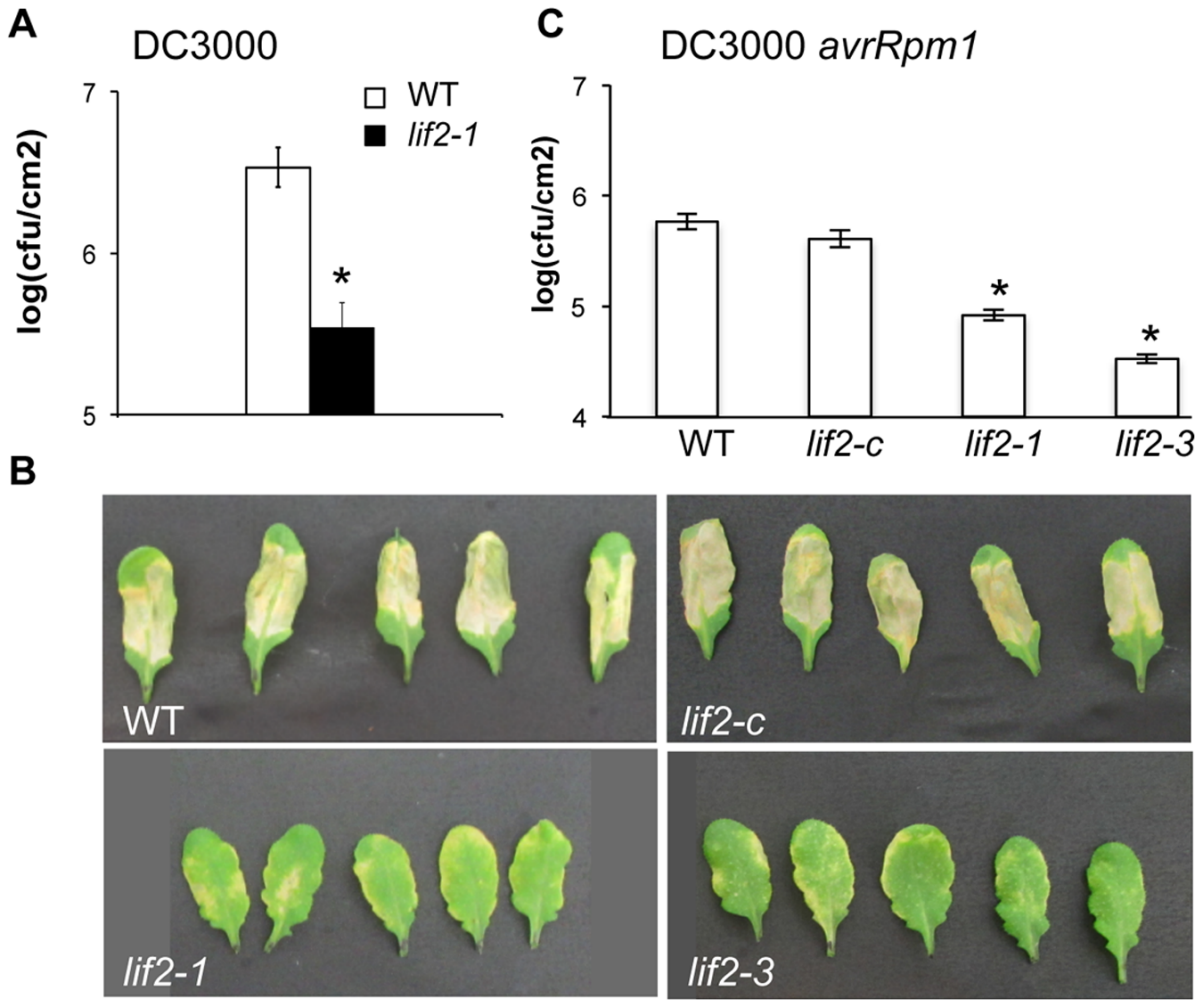
The *lif2* mutants are less susceptible to *P. syringae* infection. (A) Bacterial growth of the virulent DC3000 strain in wild-type (WT) and *lif2-1* rosette leaves at 24 hours post-inoculation (hpi). (B) Rosette leaves imaged 5 days post-inoculation (dpi) with the virulent DC3000 strain. The *lif2-1* and *lif2-3* plants had similar responses, whereas the complemented *lif2-1* mutant (*lif2-c*) behaved similarly to WT plants. Four independent experiments were performed with similar results. (C) Bacterial growth of the avirulent DC3000 *avrRpm1* strain in rosette leaves at 24 hpi. For bacterial growth experiments, each data point represents the mean value from at least thirty leaves. Similar results were obtained in two independent experiments. The bars represent standard deviation. (Student's t-test, * p<0,05).

As JA- and SA-defense-related gene expression were altered in the *lif2* mutant (Fig. 1A-B), we investigated the response of *lif2* to inoculation with the hemi-biotrophic bacterial pathogen *P. syringae* [28]. The bacterial growth of the virulent DC3000 strain was 10 times lower in *lif2-1* leaves than in wild-type leaves at 24 hours post-inoculation (hpi) (Fig. 2A). Consistently, the disease symptoms were reduced on the leaves of two independent lines, *lif2-1* and *lif2-3,* whereas severe symptoms were observed on the leaves of wild-type plants five days post-inoculation (dpi) (Fig. 2B). As expected, the *lif2-c* line, a *lif2-1* mutant line complemented with *LIF2* under the control of its own regulatory regions, was as susceptible as the wild-type plant (Fig. 2B). Similar results were obtained with the avirulent DC3000 *avrRpm1* strain (Fig. 2C). These data are consistent with the observation that SA-related genes are constitutively expressed in the *lif2* mutant.

**Figure 3:**
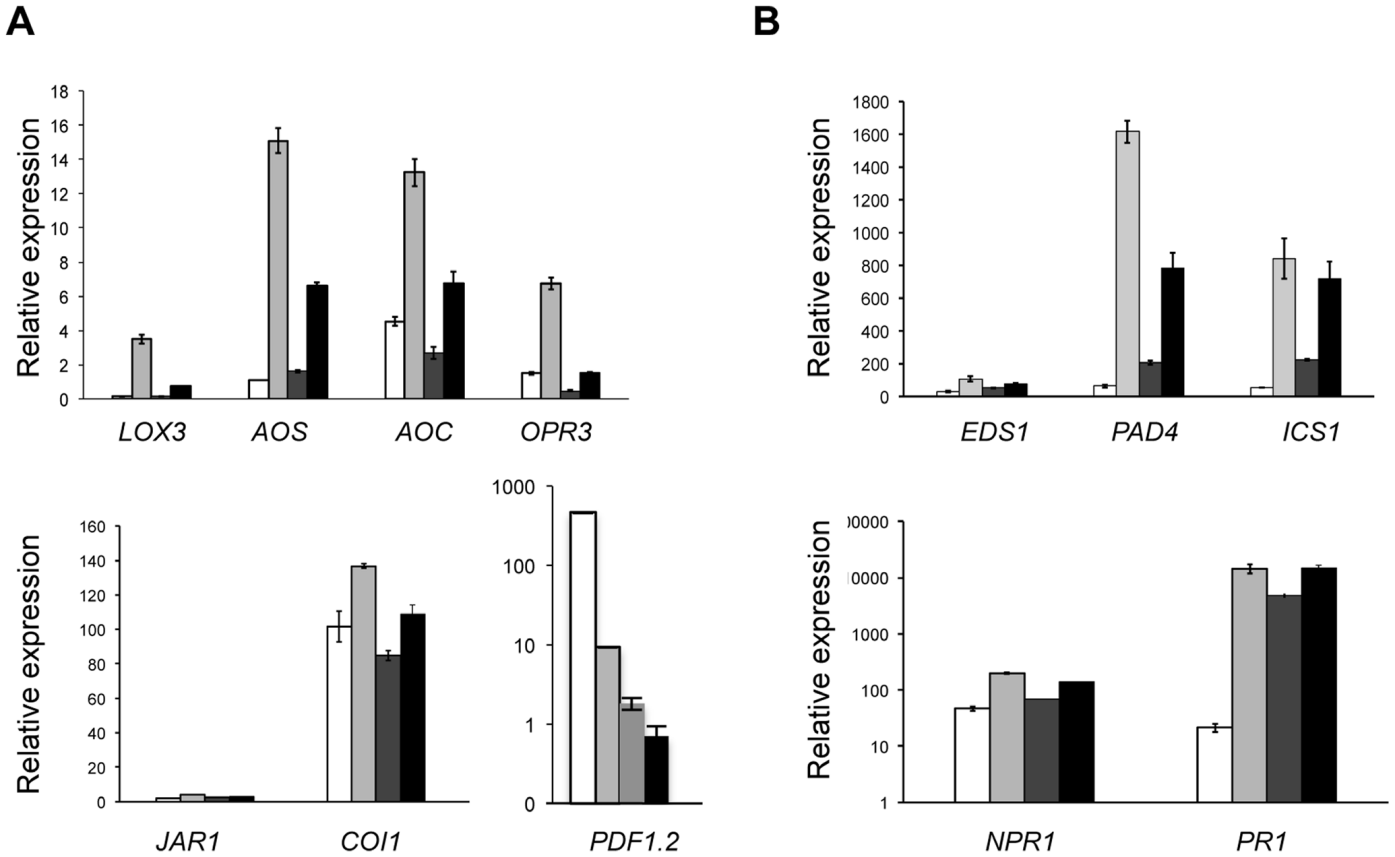
Expression of genes involved in the JA- and SA-dependent signaling pathways in response to *P. syringae* inoculation. Seven-week-old rosette leaves of wild-type (WT) and *lif2-1* mutant plants were inoculated with buffer (mock) or with the bacterial pathogen DC3000. Leaves were collected at 24 hpi and gene expression was assessed by quantitative RT-PCR. The expression of genes involved in (A) JA- and (B) SA-dependent signaling pathways is shown. WT mock (white), WT DC3000 (light grey), *lif2-1* mock (grey), and *lif2-1* DC3000 (black).

In response to *P. syringae* infection, the JA-related biosynthesis genes were upregulated in wild-type plants and to a lesser extent in *lif2-1* (Fig. 3), with the exception of *JAR1*, which was not induced. *COI1*, which encodes a component of the JA-receptor complex interacting with the bacterial phytotoxin coronatine, was upregulated in both genotypes (Fig. 3A). *PDF1.2* was downregulated in wild-type plants and not activated upon infection in *lif2*. In contrast, genes involved in SA biosynthesis and signaling pathways were upregulated in both wild-type and *lif2* plants upon DC3000 inoculation (Fig. 3B). *PAD4* induction was weaker in *lif2* than in wild-type plants, whereas *PR1* expression was similar in the mutant and wild-type plants upon *P. syringae* infection. Therefore, despite a significant basal activation in the absence of pathogen, *lif2* mutant plants were able to further activate SA-related defense genes in response to pathogen attack, and the response of the *lif2* mutant to *P. syringae* infection differed from the wild type only in the level of defense gene expression. Together, these results indicate that LIF2 is not necessary for the activation of SA- and JA-related defense genes.

### Response of the *lif2 sid2* double mutant to the biotrophic *P. syringae* pathogen

Since neither the high level of *PR1* expression in the *lif2* mutant nor the reduced susceptibility of *lif2* to *P. syringae* infection were correlated with an increase in free SA content, we hypothesized that LIF2 may act downstream of SA production. To test this hypothesis, we crossed the *lif2* mutant with the *salicylic acid induction deficient2* (*sid2/ics1*) mutant.

**Figure 4:**
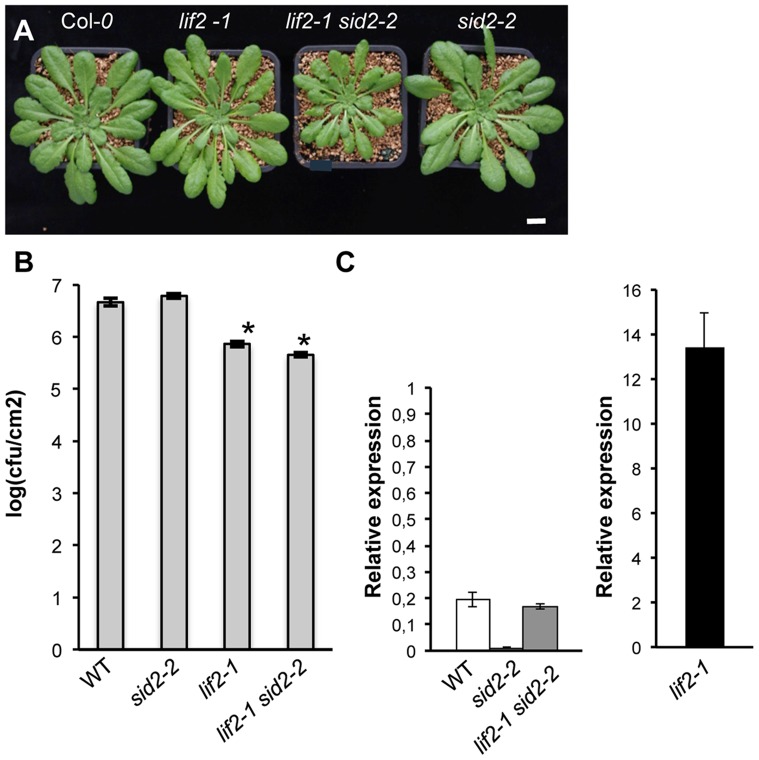
The response of the *lif2-1 sid2-2* double mutant to *P. syringae* inoculation. (A) Seven-week-old plants grown in short-day conditions. Scale bar, 1 cm. (B) DC3000 bacterial growth at 24 hpi. Each inoculated leaf was ground in MgCl_2_ and the bacterial suspension was then diluted and plated on solid medium. Twenty-four leaves were analysed per genotype. Stars indicate a significant difference from wild-type (WT) plants (Mann and Whitney, * p<0.05). Experiments were repeated twice and gave similar results. (C) The relative expression of *PR1* in untreated rosette leaves of seven-week-old plants.

The rosettes of *sid2 lif2-1* double mutant plants were smaller than those of the two parental lines, *sid2-2* and *lif2-1*, in both short-day (SD) and long-day (LD) conditions (Fig. 4A and S2). Interestingly, in SD conditions, similarly to *lif2-1* plants, the double mutant was early flowering (Fig. S2) and produced indeterminate ovaries (IDO) with an ectopic inflorescence, suggesting that the *lif2-1* mutation is epistatic to *sid2-2.*

We then investigated the response of the *lif2-1 sid2-2* double mutant to pathogens. Bacterial growth of the DC3000 strain was reduced at 24 hpi (by about 10-fold) in the *lif2-1 sid2-2* rosette leaves compared with those of the wild type (Fig. 4B), similarly to *lif2-1*. These results suggest that the reduced susceptibility of *lif2* is independent of SA biosynthesis. Interestingly, the level of *PR1* expression in *lif2-1 sid2-2* was similar to that of the wild type (Fig. 4C), suggesting that the decreased susceptibility of *lif2* may involve an *SA*-independent defense pathway.

### Response of the *lif2* mutant to a necrotrophic fungal pathogen

**Figure 5:**
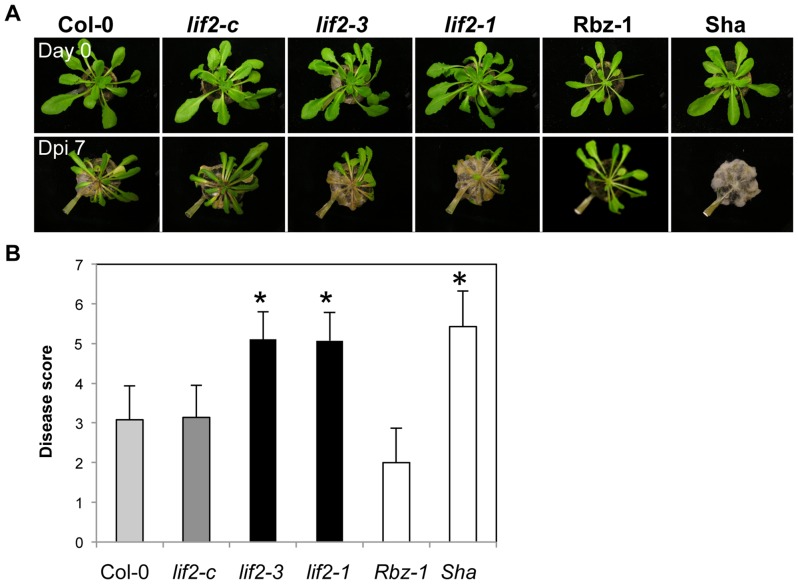
LIF2 is involved in the plant defense response to *S. sclerotiorum*. Leaves of four-week-old plants were inoculated with *S. sclerotiorum* strain S55. The *lif2* alleles and the complemented *lif2-c* line in the Col-0 background were analysed. *A. thaliana* Col-0, Rubezhnoe-1 (Rbz-1) (more resistant than Col-0), and Shahdara (Sha) (more susceptible than Col-0) accessions were used as controls. (A) Symptoms at 7 dpi. (B) The disease score was evaluated for each line at 7 dpi. Means and standard deviations were based on at least twenty plants per line. A significant difference in susceptibility relative to that of the Col-0 accession is indicated with an asterisk (Kruskal and Wallis's test, * p<0.05).

Since responses to pathogens are usually largely dependent on the pathogen lifestyle, we then assessed the defense response of *lif2* to the necrotrophic fungal pathogen *S. sclerotiorum* (strain S55). We used two *A*. *thaliana* accessions as controls, Rubezhnoe-1 (Rbz-1) and Shahdara (Sha), which were previously shown to be resistant and susceptible to *S. sclerotiorum*, respectively [29]. After inoculation, the symptoms were stronger in *lif2* than in wild-type plants (Fig. 5A and B), revealing that *lif2* is susceptible to *S. sclerotiorum*. This observation is consistent with the constitutive downregulation of JA-related genes in *lif2* reported in this study, and the finding that the JA pathway is essential for resistance to this pathogen [29], [30], [31].

**Figure 6:**
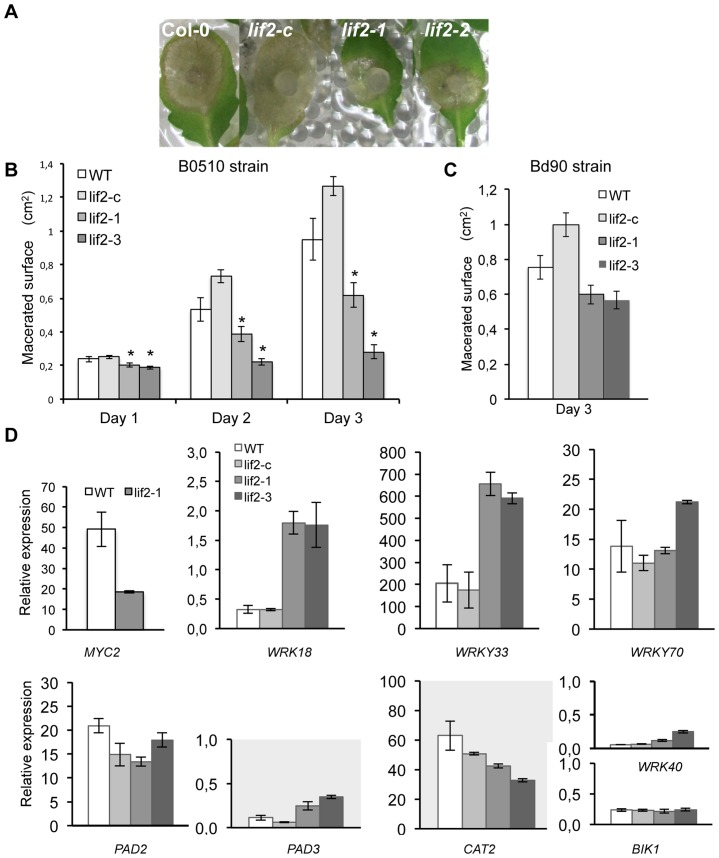
LIF2 is involved in the plant's susceptibility to *B. cinerea*. (A-C) Six-week-old plants were infected with a mycelium plug of the virulent *B. cinerea* B0510 (A-B) and BD90 (C) strains. (A) Symptoms at 3 dpi with the B0510 strain. (B-C) Lesion diameters were measured at 1 to 3 dpi. Stars indicate a significant difference from wild-type leaves on the corresponding day (Mann and Whitney's test, with a p-value of <0.05). (D) Expression of marker genes involved in the defense response to *B. cinerea*.

We then inoculated these plants with a second necrotrophic fungal pathogen, *B. cinerea.* Unexpectedly, we observed that *lif2* was less susceptible to two virulent strains of *B. cinerea,* the wild-type virulent B0510 and Bd90 strains, the latter of which was less virulent (Fig. 6A-C). These data suggest that the immune response of *lif2* is independent of the pathogen's lifestyle.

To decipher the molecular basis for the resistance of *lif2* to *B. cinerea*, we assessed the expression of a number of regulators known to be involved in disease resistance to *B. cinerea* (Table 2). For instance, mutations in the *MYB46* transcription factor gene [32], *ATGXRS13* [33], or the basic helix-loop-helix *MYC2* transcription factor [34] led to increased resistance to *B. cinerea*. In our transcriptome data, *ATGXRS13* was upregulated in *lif2*, while *MYB46* was not affected, suggesting that these two genes are probably not involved. However, we found that *MYC2* was downregulated in *lif2* (Fig. 6D), which is in agreement with the decreased susceptibility of *lif2* to *B. cinerea*. We also evaluated the expression profiles of a few other candidate genes whose downregulation led to an increased susceptibility to *B. cinerea* (i.e., *PAD2*, CYP71B15/*PAD3*, *BIK1*, *WRKY18, WRKY33*, *WRK40,* and *WRKY70*) [35], [36], [37], [38], [39], [40]. Interestingly, only the expression of *WRKY18* and *WRKY33* was significantly different in the two mutant alleles compared with wild-type or *lif2-c* plants. The loss-of-function of *CATALASE 2* (*CAT2*) triggers pathogen defense responses and resistance [41]. Furthermore, the inhibition of NADPH oxidase and of other flavoprotein enzymes involved in oxidative stress limits *B. cinerea* infection [42]. We found that *CAT2* was downregulated in *lif2,* which is in agreement with the reduced susceptibility of this mutant to *B. cinerea*. Thus, *CAT2* and the *MYC2*, *WRKY18*, and *WRK33* transcription factors are interesting candidate proteins that participate in the *lif2* response to *B. cinerea*.

**Table 2:** Genes involved in susceptibility to *B. cinerea*.

Name	AGI	Mutant phenotype	Reference
*GRXS13*	AT1G03850	Decreased susceptibility	La Camera et al. 2011
*MYC2*	AT1G32640	Decreased susceptibility	Lorenzo et al. 2004
*MYB46*	AT5G12870	Decreased susceptibility	Ramirez et al. 2011
*BIK1*	AT2G39660	Increased susceptibility	Veronese et al. 2006
*COI1*	AT2G39940	Increased susceptibility	Thomma et al. 1999
*JAR1*	AT2G46370	Increased susceptibility	Thomma et al. 1999
*PAD2*	AT4G23100	Increased susceptibility	Ferrari et al. 2003
*PAD3*	AT3G26830	Increased susceptibility	Ferrari et al. 2003
*WRKY18*	AT4G31800	Increased susceptibility*	Xu et al. 2006
*WRKY40*	AT1G80840	Increased susceptibility*	Xu et al. 2006
*WRKY33*	AT2G38470	Increased susceptibility	Zheng et al. 2006
*WRKY70*	AT3G56400	Increased susceptibility	AbuQamar et al. 2006

### Glucosinolate content is altered in the *lif2* mutant

**Figure 7:**
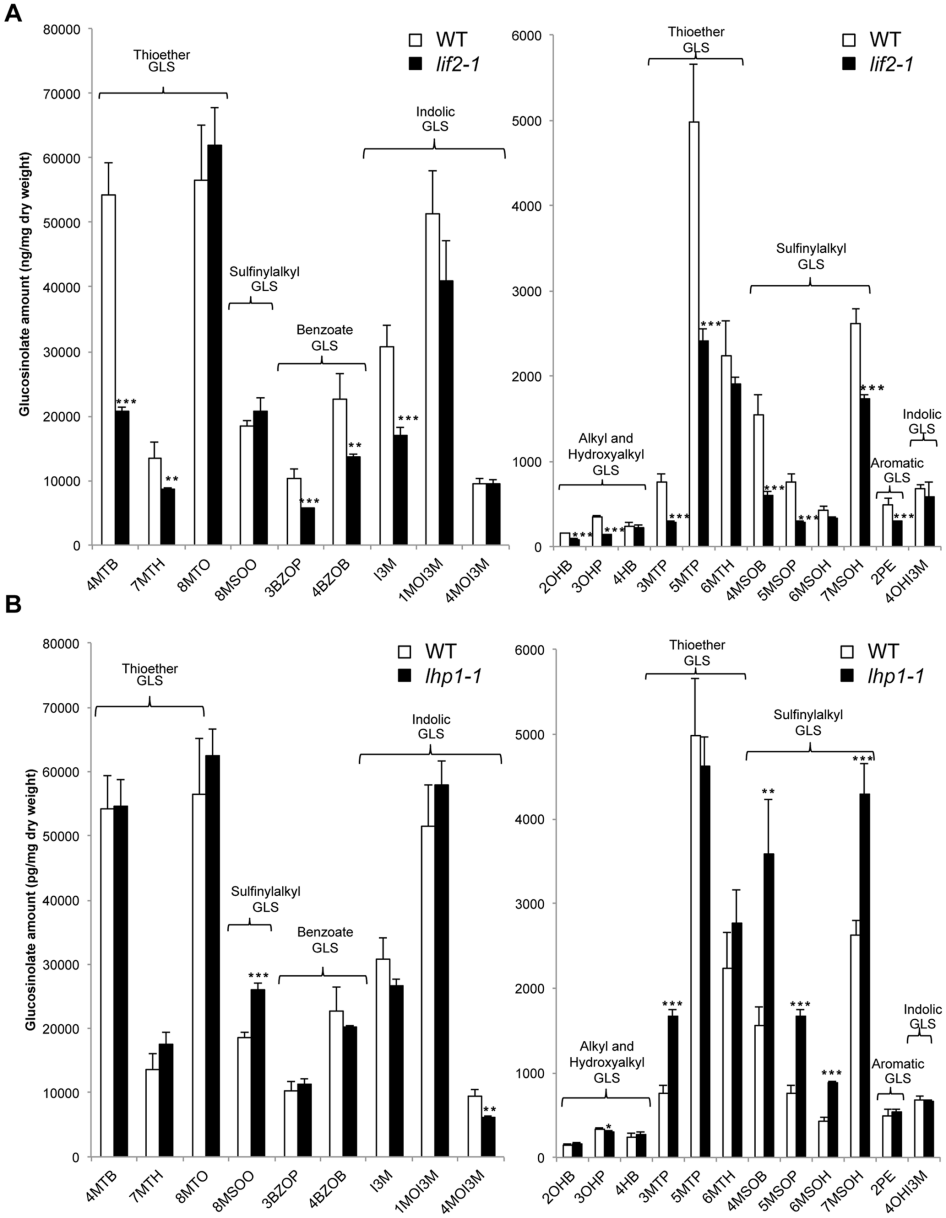
Glucosinolate (GLS) contents in *lif2-1* and *lhp1-1* young seedlings. The GLSs were quantified in *lif2-1* (A) and *lhp1-1* (B) using ESI-HPLC-MS. The full names of the GLSs are listed in Table S1.

Due to the observed enrichment in the GO term “glycosinolate metabolic process” (GO:0019757) in the *lif2-1* transcriptome (Table 1), and to the established role of glucosinolates (GLSs) and their breakdown products in plant defense [43], we extracted and quantified twenty-one of these secondary metabolites (β-thioglucoside-N-sulfated oximes), belonging to three main GLS families (aliphatic, indolic, and benzoate GLSs) in mutant and wild-type seedlings (Table S1). The GLSs were analyzed by negative electrospray ionization liquid chromatography coupled with mass spectrometry (ESI-HPLC-MS). Globally, the GLS content was lower in the *lif2* mutant than in wild-type plantlets, with decreases observed in fourteen GLSs belonging to all three main GLS families (Fig. 7A). The levels of indol-3-ylmethyl glucosinolate (I3M), 4-methylsulfinylbutyl glucosinolate (4MSOB), 4-methylthiobutyl glucosinolate (4MTB), and 5-methylthiopentyl glucosinolate (5MTP) were significantly decreased in the *lif2* mutant. Interestingly, the indolic GLS family was less affected by the *lif2* mutation, with only a decrease in indol-3-ylmethyl glucosinolate (glucobrassicin, I3M) being observed. As a control, we quantified the GLS content in the *lhp1* mutant (Fig. 7B), which is known to have an altered glucosinolate profile [15]. In *lhp1*, significantly increased levels were observed for six GLSs (i.e., 8MSOO, 3MTP, 4MSOB, 5MSOP, 6MSOH, and 7MSOH), whereas only one GLS, the indolic 4MOI3M GLS, had decreased levels relative to the wild type. Similar results were obtained using *lhp1* seeds [15]. Next, we quantified the levels of raphanusamic acid (RA), an important breakdown product of GLS that forms during the biotic stress response [44]. PEN2 myrosinase catalyzes the formation of RA from the I3M or 4MOI3M substrates. We observed a significant reduction in RA in both *lif2* (0.91 ng/mg, n = 7–8, p<0.01) and *lhp1* (0.89 ng/mg, n = 7–8, p<0.001) compared with wild-type plants (1.39 ng/mg).

Therefore, *lif2* has a GLS profile that is distinct from that of wild-type and *lhp1* seedlings. Thus, the control of GLS metabolism seems to be dependent on LIF2 and partially independent of the LHP1/LIF2 interaction [12]. Alterations to the GLS pathways may also contribute to the responses of the *lif2* mutant to pathogens.

### Molecular signature of the *lif2* mutant

**Figure 8:**
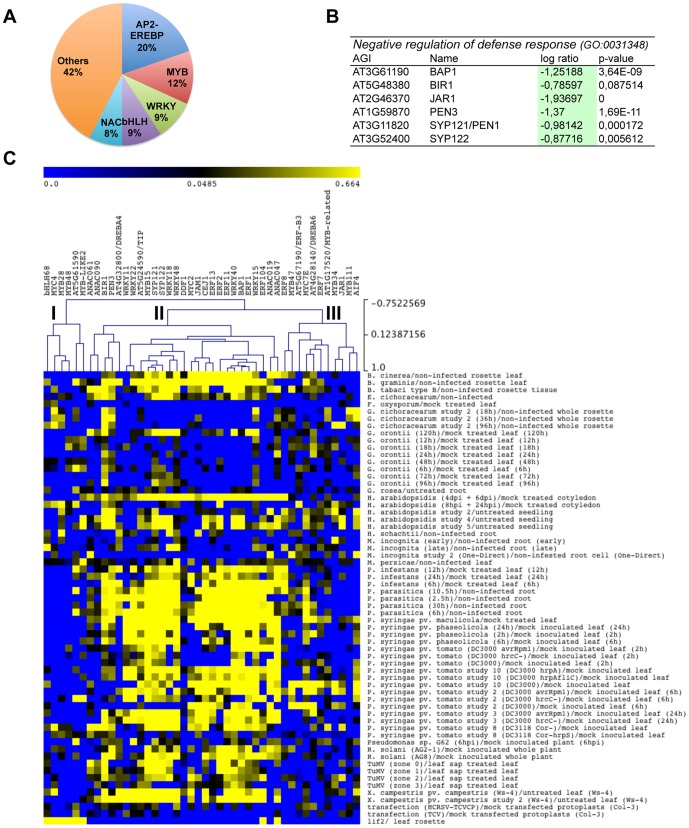
Stress-response genes deregulated in the *lif2* transcriptome. (A) The distribution of deregulated TFs in *lif2*. (B) Deregulated genes belonging to the GO term “Negative regulators of defence response”. The log ratio and the p-value were extracted from our CATMA transcriptome data. (C) Hierarchical clustering analysis performed using the MultiExperiment Viewer application, the Pearson correlation as current metric, and complete linkage clustering as the linkage method. The expression profiles of 38 deregulated TFs and 6 negative regulators in different biotic conditions were used in this analysis. Three gene clusters (I to III) were identified.

In an attempt to identify the key genes involved in the *lif2* response to biotic stress, we further analyzed the expression of transcription factors (TFs), due to their prominent role in regulating gene expression. Among the 66 TFs deregulated in the *lif2-1* transcriptome [12], we noticed that the APETALA2/ethylene-responsive element-binding protein (AP2/EREBP), WRKY, MYB, and basic helix-loop-helix (bHLH) transcription factor families were overrepresented (Table S2) (Fig. 8A). We also noticed a strong bias towards downregulation of genes of these families (Fig. 1C). For example, 11 of the 13 deregulated genes of the AP2-EREBP family were downregulated in *lif2* (Fig. 8). Out of the four subfamilies of AP2/EREBP (i.e., AP2, RAV (related to ABI3/VP1), dehydration-responsive element-binding protein (DREB), and ERF) [45], [46], only the two largest ones, the DREB and ERF subfamilies, were deregulated in the *lif2-1* mutant. The plant-specific WRKY transcription factors are key regulators of stress and plant immune responses [47], whereas the NAC TFs are involved in both development and the abiotic and/or biotic stress responses. Two stress-responsive NACs (SNACs) that were recently described [48], [49] were deregulated in the *lif2-1* transcriptome. In the MYB and bHLH families, *MYC2*, which encodes a key defense transcription factor involved in JA responses, was deregulated, (Fig. 6D) as well as the *JA-ASSOCIATED MYC2 LIKE1* gene (*JAM1*).

Furthermore, we noticed that 6 genes associated with the GO term “negative regulators of defense response” (GO:0031348) were downregulated in the *lif2-1* transcriptome. These genes might participate in the *lif2* stress response (Fig. 8B). Among these genes, JAR1 represses the SA-dependent signaling pathway; PEN3, which is an ABC transporter, restricts pathogen proliferation in the hosts; and BAP1 is a negative regulator of programmed cell death and is involved in membrane trafficking in response to external conditions [50]. These data suggest that LIF2 affects a variety of defense-related pathways.

The 38 TFs that were both deregulated in the *lif2-1* rosette transcriptome (Table S2) and belong to the AP2/EREBP, WRKY, NAC, MYB, or bHLH family, as well as the 6 negative regulators (Fig. 8B) represent a sort of molecular signature of the *lif2* mutant phenotype. We thus wondered how these 44 genes were deregulated in wild-type genetic backgrounds in response to biotic stress. To address this question, we selected 62 transcriptome profiles from plants treated with pathogens compared with those that were not, in the Genevestigator database [51] and extracted the expression of these 44 genes. We then performed a hierarchical clustering analysis using of the MultiExperiment Viewer application [52], and identified three main gene clusters, which are characterized by specific co-expression profiles of *MYC* and *MYB* (Fig. 8C). The small Cluster I (containing *MYC4*/*MYB28*/*MYB48*) contained genes that are upregulated in *lif2-1* and downregulated in response to most pathogen treatments. The large Cluster II (containing *MYC2/MYB15*) contained genes downregulated in *lif2-1* and up- or downregulated in response to pathogen treatments, with no obvious link to the biology of the pathogens. Finally, Cluster III (containing *MYC7E*/*MYB34*) mainly contained genes repressed in *lif2-1* and in response to most biotic stresses, except in response to *P. syringae*, and thus, which are probably associated with the JA pathway due to the presence of JAR1. In conclusion, our clustering analysis revealed that (i) the expression profiles of the 44 selected genes involved in the response to pathogens were diverse, (ii) the *MYC* and *MYB* genes were co-expressed in different combinations, and (iii) that *lif2* exhibited a unique expression pattern, with opposite profiles to most of the 62 analyzed transcriptome profiles, which might be in line with its specific response to pathogens.

## Discussion

Plants have evolved both constitutive and induced defense mechanisms to counteract pathogen attacks. Most of the induced mechanisms are based on the production of a complex repertoire of plant metabolic compounds and signaling hormones, which activate appropriate defense pathways, based on the nature of the pathogens [53], [54]. Salicylic acid, a phenolic phytohormone, and the jasmonic acid phytohormone, a polyunsaturated fatty acid derived from α-linolenic acid, are two key molecules in plant defense mechanisms [55]. SA, which also possesses medicinal properties [24] and plays a central role in animal immunity [56], and its derivatives, have crucial roles in defense against biotrophic pathogens [57]. They also regulate various aspects of abiotic stress responses, plant growth, and development. The JA and SA defense signaling pathways have been shown to cross-communicate and to be mostly antagonistic, providing plants with a regulatory potential to fine-tune their defense reaction depending on the type of pathogen encountered [22], [23]. SA and JA are mainly involved in the response to biotrophic and necrotrophic pathogens, respectively. However, complex crosstalk exists between the hormone signaling pathways involved in plant responses to pathogens [26]. Secondary metabolites, such as glucosinolates, are also involved in the biotic stress response in plants [43].

Only a few RBPs have emerged as regulators of the biotic stress response in plants [8], [58], [59], [60]. For instance, AtRBP-DR1, a putative component of the resistance protein RPS2 complex, positively regulates defense responses mediated by SA in *A. thaliana* [61]. The conserved MOS2 protein, a putative RBP with both G-patch and KWO domains, is required for resistance against *P. syringae pv. maculicola* ES4326 and *Peronospora parasitica* Emoy2 [62], whereas overexpression of the *Gossypium hirsutum* GhZFP1 protein, which contains a CCCH-type zinc finger present in some RBPs, has been shown to enhance both disease resistance to the *Rhizoctonia solani* fungus and salt stress tolerance [63]. Over-expression of the *Capsicum annuum* RNA-binding protein 1, CaRBP1, in *A. thaliana* conferred reduced susceptibility to infection by the biotrophic oomycete *Hyaloperonospora arabidopsidis* [9]. Except for the hnRNP AtGRP7 [9], the RBP-mediated defense mechanisms remain poorly understood.

Here, we studied the role of *A. thaliana* RBP LIF2 in the plant's response to biotic stress. We demonstrated that *LIF2* loss-of-function results in pathogen resistance, in a manner that is independent of the pathogen's lifestyle or infection strategy. Indeed, the *lif2* mutant was less susceptible to both the hemi-biotrophic bacterial *P. syringae* pv. *tomato* pathogen and the necrotrophic ascomycete *B. cinerea*, two pathogens with substantial impacts on agronomical production due to their wide range of host specificities [17], [18]. However, *lif2* was more susceptible to the necrotrophic ascomycete *S. sclerotiorum*, which attacks more than 400 plant species around the world.

The three pathogens used in this study induce different plant defense pathways and their associated defense molecules: the JA and ethylene pathways, and possibly the SA pathway, are activated in response to *S. sclerotiorum* infection [29], [30]; the SA pathway is key in the plant's response to *P. syringae* infection; and JA and camalexin are involved in the response to *B. cinerea* infection [64]. Our analysis of the *lif2* mutant revealed that SA-related genes (*ICS1/SID2*, *PR1*) were constitutively activated. The upregulation of *PR1* was in agreement with the decreased susceptibility of *lif2* to *P. syringae*, and this response was also observed in other mutants with reduced susceptibility to *P. syringae*, such as *cpr1* and *cpr5* [65]. However, the constitutive activation of the SA biosynthesis pathway did not lead to an overaccumulation of free SA in the *lif2* mutant. SA undergoes various chemical modifications, which affect its activity, catabolism, transport, and storage [66]. The accumulation of free SA is fine-tuned by the transcriptional regulation of genes involved in SA biosynthesis, but also by the modulation of enzymes that modify SA [24]. Our data suggest that genes of the SA pathway may undergo post-transcriptional regulation or that a homeostasis mechanism may control free SA accumulation and maintain it at wild-type levels in *lif2*. Furthermore, the SA production pathway is not fully elucidated in *A. thaliana,* since no isochorismate pyruvate lyase, which is required to convert isochorismate into SA in bacteria, has been identified to date. A second SA biosynthesis pathway, which is regulated by phenylalanine ammonia lyase (PAL), has been proposed to exist in plants [57], [67]. In *lif2*, the discrepancy between the constitutive upregulation of the SA/ICS1-dependent pathway and the lack of SA accumulation may also suggest some crosstalk between the ICS1 and PAL pathways to fine-tune SA production. Finally, the *lif2 sid2* double mutant was less susceptible than wild-type plants to *P. syringae*, despite exhibiting a *PR1* expression level similar to that of wild-type plants. Therefore, the reduced susceptibility of the *lif2* mutant to *P. syringae* was independent of SA in non-challenged plants, suggesting that LIF2 participates in plant defense *via* a novel defense pathway that is independent of the SA-signaling defense pathway.

Furthermore, we showed that the GLS profile of the *lif2* mutant was altered. These changes might indirectly participate in the increased resistance phenotype of *lif2*. Indeed, GLSs function in the plant's defense response to herbivores and fungal pathogens [44], [68], [69], [70]. Ward *et al.* (2010) suggested that GLSs do not play a direct role in the response to bacteria, but possibly have an intermediary role by influencing some defense pathways. Indeed, after infection of *A. thaliana* with *P. syringae* DC3000, a significant reduction in I3M and 4MOI3M was observed [71]. In the *lif2* mutant, a low level of I3M was observed, but no change was observed in 4MOI3M in the absence of bacterial challenge. The global decrease in GLS and RA contents observed in *lif2* might thus contribute to the control of the basal defense pathways. Interestingly, JA is a regulator of GLS gene expression and of GLS accumulation via multiple pathways [72], [73].

**Figure 9:**
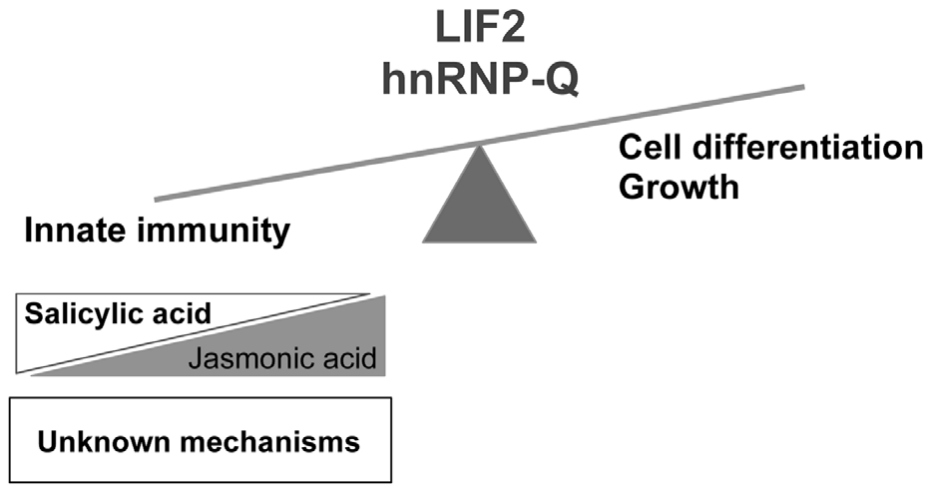
Model for the functions of the hnRNP-Q LIF2 protein. LIF2 may regulate the balance between development and plant immunity by minimizing the energy cost of plant defense.

Several TFs have recently been shown to be involved in GLS gene regulation, including *MYB28*, *MYB29*, and *MYB76*, which are key regulators of the aliphatic-GLSs, and *MYB34* and *MYB51*, which are regulators of the indole-GLSs. Furthermore, MYC2, MYC3, and MYC4 can form protein complexes with all known GS-related MYBs to regulate GLS biosynthesis [74]. In the *lif2* mutant, *MYB28* is downregulated, whereas *MYB34* is upregulated, and the levels of members of the two GLS families were decreased. However, *MYC2* is downregulated in the *lif2* mutant. Furthermore, several TFs related to defense responses are deregulated in *lif2*. WRKY33 is an essential transcription factor in the defense against *B. cinerea* that acts by controlling the expression of genes involved in redox homeostasis, SA signaling, and camalexin biosynthesis and thus affecting the SA-JA balance [40]. The activation of *WKRY33* observed in *lif2* may have downstream effects, which may play a role in the resistance of *lif2* to *B. cinerea.* Therefore, it is likely that multiple components of different defense pathways contribute to the primed state of *lif2* in the absence of pathogen, and to its reduced pathogen susceptibility. Interestingly, LIF2 interacts with the chromatin-associated protein LHP1, a subunit of the Polycomb Repressive Complex, which interacts with numerous genomic sites and regulates their expression [12], [14]. Some defense-related genes are present among the LHP1 targets. Whether LIF2 acts coordinately with LHP1 at these defense-related loci to control them constitutes an interesting research question, as the role of chromatin proteins in plant immunity is poorly documented [75], [76], [77], [78], [79]. For instance, PIE1, a member of the SWR1 subfamily, was shown to negatively regulate plant defense [80], whereas loss-of function of the SIRTUIN2 histone deacetylase, a homolog of the yeast Silent information repressor 2 (Sir2) protein [81] and of SDG8 histone methyltransferase [82] alter plant-pathogen responses. It was proposed that chromatin modifications may participate in defense priming in plants [83]. Thus, our study highlights an emerging role for the LIF2 chromatin-associated protein in biotic stress responses, with a putative suppressor function in plant immunity (Fig. 9).

Finally, LIF2 is also involved in the maintenance of growth and cell determination during floral development [12]. Therefore, our study illustrates the trade-off existing between plant development and plant defense against various enemies in a changing environment [84], [85]. LIF2 may thus regulate the balance between development and plant immunity by limiting the cost associated with the plant defense response (Fig. 9). It was suggested that priming of SA-related defense responses greatly enhances disease resistance and plant fitness, but diminishes fitness in the absence of pathogens [85]. Furthermore, since both developmental and plant defense processes require adaptation to environmental conditions, sharing common elements with dual functions may allow for better fine-tuning [86]. Since the human hnRNP-Q homolog of LIF2, SNAP1/SYNCRIP, also acts as a cellular RBP and as a suppressor of human immunity against virus infection, our data highlight a conserved role for hnRNP-Q in eukaryote immunity. The dual function of hnRNP-Q proteins in development and defense is likely to involve key conserved molecular events in eukaryotic cells. Identifying the associated RNA partners of LIF2 RBP may elucidate the underlying mechanism.

## Materials and Methods

### Plant materials

*Arabidopsis thaliana* lines used in this study were in the Columbia (Col-0) accession. The homozygous *lif2*-1 and *lif2-3* mutants were described in [12]. The *lif2-c* line is a *lif2-1* mutant complemented with the *LIF2* genomic region under the control of a 3-kb promoter region. The *sid2-2* [87] mutant was crossed with the *lif2-1* allele. Double mutants were selected by PCR using specific primers (Table S3, [12]).

For *P. syringae*, *B. cinerea,* and *S. sclerotiorum* pathogen response analyses, plants were grown on soil in growth chambers, under controlled short-day conditions (8 hours light/16 hours dark), at 20°C and 70% hygrometry.

### Primers

All primers are described in Table S3.

### Bacterial inoculation

The virulent strain *P. syringae* pv. tomato DC3000 (*Pst* DC3000) [88] and the avirulent strain *P. syringae* pv. tomato DC3000, carrying the avirulent gene *avrRpm1* (*Pst* DC3000 *avrRpm1*) [28], were grown at 28°C on King's B solid medium plates containing the appropriate antibiotics. Briefly, a fresh bacterial suspension was scraped off the growth plate, and resuspended in 10 mM MgCl_2_ at an OD600 of 0.01 (at a concentration of 10^6^ cfu ml^−1^). The bacterial suspension was infiltrated into the abaxial side of 24 to 30 rosette leaves of seven-week-old plants (6 to 10 plants per genotype) using a 1-ml syringe without a needle. Control inoculations (Mock) were performed using 10 mM MgCl_2_. Bacterial growth was assessed 24 hours after infiltration. Whole leaves were homogenized in 500 µl of 10 mM MgCl_2_. After serial dilutions, homogenates were plated on King's B medium and incubated at 28°C for 2 days before colonies were counted. The surface area of each leaf was measured using ImageJ software in order to calculate cfu/cm^2^. Day 0 titers ranged from log_10_ = 3.6–3.9. Leaves were photographed 5 days post-infiltration (dpi). All experiments were repeated at least twice with similar results.

### Fungal pathogen inoculation procedure

Four-week-old plants were inoculated with discs carrying *S. sclerotiorum* (S55 strain) mycelia, as previously described [29]. Plant inoculations were performed in a Helios 1200 Phytotron under a light period of 9 h at 22°C and 80% relative humidity during the first 3 days and 66% thereafter. At least 20 plants of each genotype were inoculated. The disease score of each line was evaluated seven days post-inoculation. Two independent experiments were performed with similar results.

Leaves of seven-week-old plants were inoculated with mycelial plugs of the wild-type strain *B. cinerea* B0510 and Bd90 (diameter of 3 mm), as previously described [89]. Infected leaves were placed in a Petri dish under high humidity and incubated at 21°C. Lesion surfaces were measured daily for three days using ImageJ software.

### Quantification of hormonal contents

Rosettes of seven-week-old plants were frozen immediately after harvest and ground in liquid nitrogen. Four pools of 6 rosettes were collected. Frozen material (100 mg) was extracted with 3 ml of acetone/water/acetic acid (80/19/1, v:v:v) containing the following stable isotope labelled internal standards: 10 ng [4-2H] ABA (NRC-CNRC Plant Biotechnology Institute, Saskatoon, Canada), 50 ng [4-2H] salicylic acid (Olchemlm, Olomouc, Czech Republic), 1 ng [5-2H] jasmonic acid (CDN Isotopes CIL Cluzeau, Sainte Foy la Grande, France), and 10 ng [6-13C] indole-3-acetic acid (Cambridge Isotope Laboratory, Andover, MA). The extract was vigorously shaken for 30 s, sonicated for 1 min at 25 Hz, shaken for 10 min at room temperature, and then centrifuged (8230 g, 4°C, 15 min). The supernatants were collected and the pellets were extracted again with 1 ml of the same extraction solution, and then vigorously shaken (1 min) and sonicated (1 min, 25 Hz). Following centrifugation, the two supernatants were pooled and dried. The dry extract was dissolved in 140 µl acetonitrile/water (50/50, v/v), filtered, and submitted to analysis by HPLC-electrospray ionisation-MS/MS (HPLC-ESI-MS/MS). The compounds were introduced into the ESI source using a Waters 2695 separation module (Alliance; Waters, Milford, MA, USA) equipped with a Waters 2487 dual UV detector. Separation was achieved on a reverse-phase column (Uptisphere C18 ODB, 150*2.1 mm, Interchim, Montluçon, France), using a flow rate of 0.15 ml min^−1^ and a binary gradient as follows: (A) acetic acid 0.1% (v/v) and (B) acetonitrile. The solvent gradient was programmed as follows: 0–5 min, 20% A; 5–15 min, 50% A; 15–30 min, 100% B; and 30–42 min, 20%. The analyses were performed on a Waters Quattro LC Triple Quadripole Mass Spectrometer (Waters) operating in a Multiple Reaction Monitoring (MRM) scanning mode. The instrumental parameters were set as follows: capillary, 2.70 kV (negative mode); extractor, 3 V; and source block and desolvation gas temperatures, 120°C and 350°C, respectively. Nitrogen was used for the nebulization and desolvation (77 L h^−1^ and 365 L h^−1^, respectively), and argon was used as the collision gas at 2.83 10^−3^ mbar. For a 5-µL injection volume of sample prepared and reconstituted in 140 µl of 50/50 acetonitrile/H2O (v/v), the limit of detection (LOD) and limit of quantification (LOQ) were extrapolated for each hormone from a calibration curve and sample using the Quantify module of MassLynx (version 4.1 software). The parameters used for MRM quantification and the LOD and LOQ are listed in Tables S4 and S5, respectively. The amount of JA was expressed as a ratio of peak areas (209>62/214>62) per fresh weight, due to impurities contained in the D5-JA standard.

### Glucosinolate extraction and quantification

Glucosinolate analyses were performed on 15-day-old *in vitro* seedlings grown under long-day conditions. The plant material was rapidly collected, frozen, and lyophilized. Lyophilized material (30–50 mg) was ground in liquid nitrogen. Then, 0.2 mg of sinigrin hydrate standard (Sigma Aldrich Ref. 85440) was added as an internal tracer for recovery and analytical purposes. The samples were extracted using 2.5 ml of extraction solution (75% acetonitrile/25% water solution), vigorously homogenized for 5 min in a Polytron homogenizer (Fischer Scientific), and centrifuged (8000 g, 20°C, 15 min). The supernatants were collected and the pellets were extracted with 1 ml of the extraction solution and sonicated (15 min, 25 Hz). Following centrifugation, the supernatant was pooled with the first supernatant. The extracts were then evaporated to dryness using a Thermo Savant Speedvac overnight, at ambient temperature. A water/acetonitrile solution (95/5) was added to the dried extracts, which were filtered and diluted to the third with water/acetonitrile (95/5), prior to negative electrospray LC-MS analysis on an Alliance 2695 system coupled to Quattro LC (Waters). Glucosinolates were identified by retention time, mass, isotopic pattern, and fragment ions [90], [91]. The concentrations of the metabolites of interest were quantified using the sinigrin response, as previously described [92]. Three independent extraction analyses were carried out per biological experiment and two to three biological replicates were performed. Biochemical information about the different classes of GLSs and their biosynthesis pathways can be found in various databases (e.g., http://www.genome.jp/kegg-bin/show_pathway?ko00966+C08417 or the AraCyc website).

### Gene expression analysis

Total RNA was isolated from various tissues, using the RNeasy Plant Mini Kit (QIAGEN), and treated with RNase-free DNaseI (Invitrogen). Reverse transcription (RT) reactions were performed with Superscript II reverse transcriptase (Invitrogen), according to the manufacturer's instructions. Quantitative real-time PCR (qPCR) was performed on Eppendorf Mastercycler® ep realplex (Eppendorf) using MESA FAST qPCR MasterMix Plus for SYBR® Assay (Eurogentec), as per the manufacturer's instructions.

## Supporting Information

Figure S1 ***LIF2*** **expression in response to** ***P. syringae*** **DC3000 and DC3000** ***avrRpm1*** **inoculations.** hpi, hour post-infection.
(PPTX)

Figure S2 **Phenotypes of the** ***lif2-1 sid2-2*** **double mutant.** (A) Rosettes of 46-day-old plants grown in long-day (LD) conditions. (B) Rosette leaves in LD conditions. (C) Rosettes of 53-day-old plants grown in short-day (SD) conditions. (D) Rosette leaves in SD conditions. (E) The *lif2-1 sid2-2* mutant is early flowering. Number of rosette leaves produced by plants grown in LD and SD conditions.
(PPTX)

Table S1 **The nomenclature of glucosinolate compounds.**
(DOCX)

Table S2 **Selection of transcription factors differentially expressed in the** ***lif2*** **transcriptome.**
(DOCX)

Table S3 **Gene-specific oligonucleotides used in this study.**
(DOCX)

Table S4 **Parameters for the LC-ESI-MS/MS analysis in negative mode.**
(DOCX)

Table S5 **The limit of detection (LOD) and limit of quantification (LOQ).**
(DOCX)
