# Supplementary material for: The hnRNP-Q Protein LIF2 Participates in the Plant Immune Response
Source: PLoS One. 2014 Jun 10;9(6):e99343. doi: 10.1371/journal.pone.0099343 (PMC4051675; doi:10.1371/journal.pone.0099343)
Supplement: Figure S1 — LIF2 expression in response to P. syringae DC3000 and DC3000 avrRpm1 inoculations. hpi, hour post-infection. (PPTX) [file pone.0099343.s001.pptx]

## Slide 1
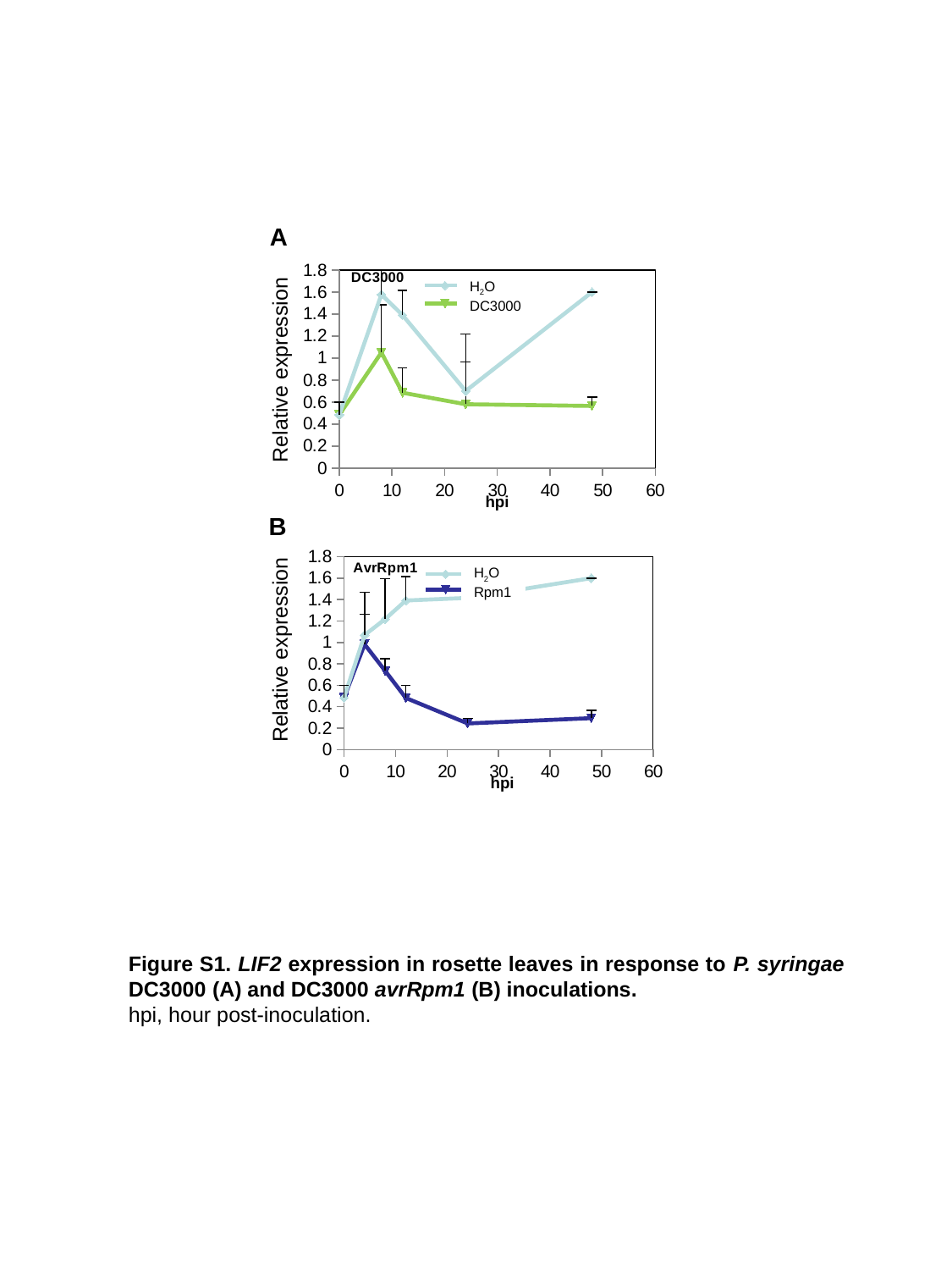

### Chart
| Category | H2O | DC3000 |
|---|---|---|Relative expression
hpi
### Chart
| Category | H2O | Rpm1 |
|---|---|---|Relative expression
hpi
A
H2O
DC3000
B
H2O
Rpm1
Figure S1. LIF2 expression in rosette leaves in response to P. syringae DC3000 (A) and DC3000 avrRpm1 (B) inoculations.
hpi, hour post-inoculation.
