# Supplementary material for: The hnRNP-Q Protein LIF2 Participates in the Plant Immune Response
Source: PLoS One. 2014 Jun 10;9(6):e99343. doi: 10.1371/journal.pone.0099343 (PMC4051675; doi:10.1371/journal.pone.0099343)
Supplement: Figure S2 — Phenotypes of the lif2-1 sid2-2 double mutant. (A) Rosettes of 46-day-old plants grown in long-day (LD) conditions. (B) Rosette leaves in LD conditions. (C) Rosettes of 53-day-old plants grown in short-day (SD) conditions. (D) Rosette leaves in SD conditions. (E) The lif2-1 sid2-2 mutant is early flowering. Number of rosette leaves produced by plants grown in LD and SD conditions. (PPTX) [file pone.0099343.s002.pptx]

## Slide 1
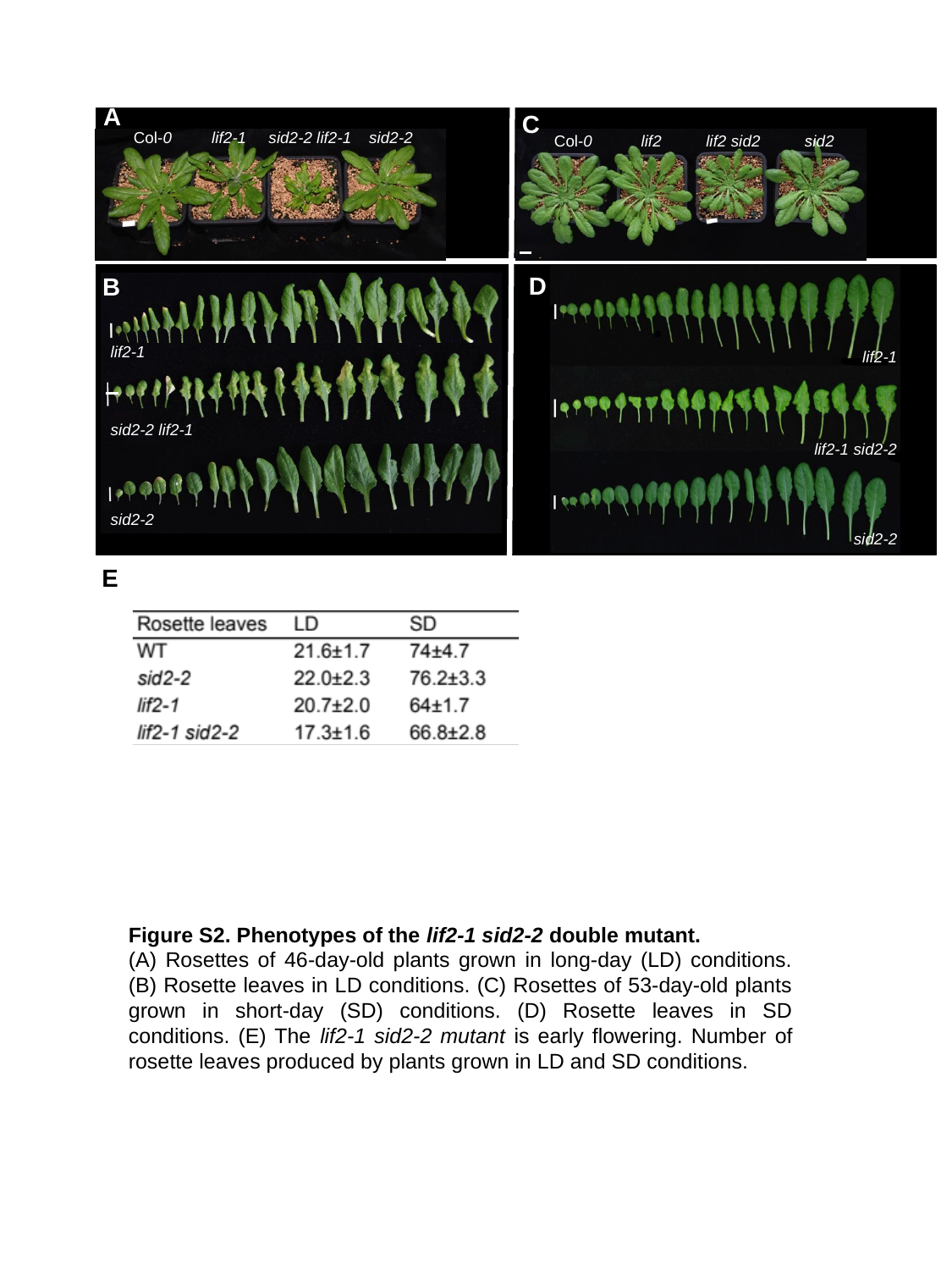

A
C
Col-0 lif2-1 sid2-2 lif2-1 sid2-2
Col-0 lif2 lif2 sid2 sid2
D
B
Col-0 lif2-1 sid2-2 lif2-1 sid2-2
 lif2-1
 lif2-1
 sid2-2 lif2-1
 lif2-1 sid2-2
 sid2-2
 sid2-2
E
 lif2-1 sid2-2
lif2-1 sid2-2
Figure S2. Phenotypes of the lif2-1 sid2-2 double mutant.
(A) Rosettes of 46-day-old plants grown in long-day (LD) conditions. (B) Rosette leaves in LD conditions. (C) Rosettes of 53-day-old plants grown in short-day (SD) conditions. (D) Rosette leaves in SD conditions. (E) The lif2-1 sid2-2 mutant is early flowering. Number of rosette leaves produced by plants grown in LD and SD conditions.
