# Supplementary material for: The hnRNP-Q Protein LIF2 Participates in the Plant Immune Response
Source: PLoS One. 2014 Jun 10;9(6):e99343. doi: 10.1371/journal.pone.0099343 (PMC4051675; doi:10.1371/journal.pone.0099343)
Supplement: Table S1 — The nomenclature of glucosinolate compounds. (DOCX) [file pone.0099343.s003.docx]

**Table S1. The nomenclature of glucosinolate compounds.**

| Symbol | Name | Common name | Biosynthesis pathway from |
| --- | --- | --- | --- |
| 2OHB | 2-hydroxy-3-butenyl glucosinolate | Progoitrin | Methionine |
| 3OHP | 3-hydroxy-4-pentenyl glucosinolate |  | Methionine |
| 4HB | 2-hydroxy-2-methylpropyl glucosinolate | Glucoconringiin | Methionine |
| 3MTP | 3-methylthiopropyl glucosinolate | Glucoiberverin | Methionine |
| 4MTB | 4-methylthiobutyl glucosinolate | Glucoerucin | Methionine |
| 5MTP | 5-methylthiopentyl glucosinolate | Glucoberteroin | Methionine |
| 6MTH | 6-methylthiohexyl glucosinolate | Glucolesquerellin | Methionine |
| 7MTH | 7-methylthioheptyl glucosinolate |  | Methionine |
| 8MTO | 8-methylthiooctyl glucosinolate |  | Methionine |
| 4MSOB | 4-methylsulfinylbutyl glucosinolate | Glucoraphanin | Methionine |
| 5MSOP | 5-methylsulfinylpentyl glucosinolate | Glucoalyssin | Methionine |
| 6MSOH | 6-methylsulfinylhexyl glucosinolate | Glucohesperin | Methionine |
| 7MSOH | 7-methylsulfinylheptyl glucosinolate | Glucoibarin | Methionine |
| 8MSOO | 8-methylsulfinyloctyl glucosinolate | Glucohirsutin | Methionine |
| 2PE | 2-phenylethyl glucosinolate | Gluconasturtiin | Phenylalanine |
| 3BZOP | 3-benzoyloxypropyl glucosinolate | Glucomalcomin | Methionine |
| 4BZOB | 4-benzoyloxybutyl glucosinolate |  | Methionine |
| I3M | Indol-3-ylmethyl glucosinolate | Glucobrassicin | Tryptophan |
| 1MOI3M | 1-methoxyindol-3-ylmethylglucosinolate | Neoglucobrassicin | Tryptophan |
| 4OHI3M | 4-hydroxyindol-3-ylmethylglucosinolate | 4-hydroxy-glucobrassicin | Tryptophan |
| 4MOI3M | 4-methoxyindol-3-ylmethylglucosinolate | 4-methoxy-glucobrassicin | Tryptophan |
