# Supplementary material for: The hnRNP-Q Protein LIF2 Participates in the Plant Immune Response
Source: PLoS One. 2014 Jun 10;9(6):e99343. doi: 10.1371/journal.pone.0099343 (PMC4051675; doi:10.1371/journal.pone.0099343)
Supplement: Table S2 — Selection of transcription factors differentially expressed in the lif2 transcriptome. (DOCX) [file pone.0099343.s004.docx]

**Table S2. Selection of transcription factors in *lif2* transcriptome.**
