# Supplementary material for: The hnRNP-Q Protein LIF2 Participates in the Plant Immune Response
Source: PLoS One. 2014 Jun 10;9(6):e99343. doi: 10.1371/journal.pone.0099343 (PMC4051675; doi:10.1371/journal.pone.0099343)
Supplement: Table S3 — Gene-specific oligonucleotides used in this study. (DOCX) [file pone.0099343.s005.docx]

**Table S3. Gene-specific oligonucleotides.**

| Name | AGI | Sequence (5' to 3') |
| --- | --- | --- |
| AOC-03 | AT1G13280 | GATTCGTTCCTTGCCATCAC |
| AOC-04 | AT1G13280 | AACCTGTCCGTAGGCACCTT |
| AOS/CYP74A-01 | AT5G42650 | GACCAATCAAAGACCGTTGG |
| AOS/CYP74A-02 | AT5G42650 | CGGATTCGTGATTTGAAGAAC |
| BIK1-F2 | AT2G39660 | CCGATCTCCGTTCACATCTC |
| BIK1-R2 | AT2G39660 | TTCAACCCACAAGGCAAAA |
| CAT2-F1 | AT4G35090 | TCCAGAGCGTGTGGTTCAT |
| CAT2-R1 | AT4G35090 | CATGAGTGACCTCAAAGAAACCT |
| EDS1-01 | AT3G48090 | AAAGGCGTCTGTAGAGGAAACTT |
| EDS1-02 | AT3G48090 | TTGGACGGAAGACTTTCTGG |
| ICS1-03 | AT1G74710 | ATTGATCTATGCGGGGACAG |
| ICS1-04 | AT1G74710 | TCAAGCTCATTCCACTCTGAAG |
| LIF2-101 | AT4G00830 | CGAGGAGGGTAGCTACAGTGA |
| LIF2-102 | AT4G00830 | TCCTCCTCTTCATACTCTTCTACTTGT |
| LOX3-01 | AT1G17420 | CACTGCTGGTGCATACGG |
| LOX3-02 | AT1G17420 | GAGGCCTTCCATGTCGAAC |
| NDR1-01 | AT5G06320 | TAAGAACACGACGGTGGTTG |
| NDR1-02 | AT5G06320 | CCGTCAAGCAGCACCAAT |
| NPR1-01 | AT1G64280 | AGGGGATATACGGTGCTTCA |
| NPR1-02 | AT1G64280 | ATGCACTTGCACCTTTTTCC |
| OPR3-05 | AT2G06050 | GGCTTTAGAAGCTTCCGAGAT |
| OPR3-06 | AT2G06050 | GAATCGCATTCAAAGCAGAAA |
| PAD2-F2 | AT4G23100 | AACTTCCCTGTCTCCCTGGT |
| PAD2-R2 | AT4G23100 | TTGTCAGATGGTTTTCCCAAT |
| PAD3-F | AT3G26830 | CCATCATCGGAAACTTACACCAA |
| PAD3-R | AT3G26830 | GGCGAGATTACGGCGATTC |
| PAD4-01 | AT3G52430 | TATGCGAATACATTGGTGACG |
| PAD4-02 | AT3G52430 | CGAGTTCTTCGCTTTAACATCC |
| PDF-10 | AT5G44420 | TGATCCATGTTTGGCTCCTTCA |
| PDF-09 | AT5G44420 | AAGCCAAGTGGGACATGGTCA |
| PR-1fw01 | AT2G14610 | CGGAGCTACGCAGAACAACT |
| PR-1rv01 | AT2G14611 | CTCGCTAACCCACATGTTCA |
| SID2-F | AT1G74710 | CAGTCCGAAAGACGACCTCGAGTT |
| SID2-R2 | AT1G74710 | GCTGGAGTTGGATGCAGAGCAGCC |
| VSP2-01 | AT5G24770 | TCAGTGACCGTTGGAAGTTGTG |
| VSP2-02 | AT5G24770 | GTTCGAACCATTAGGCTTCAATATG |
| WRKY18-1 | AT4G31800 | GTACAACGCAGCGCAGAGGAT |
| WRKY18-2 | AT4G31800 | ACCCACCCTGGCTTGTAGCAT |
| WRKY33-F | AT2G38470 | GGGAAACCCAAATCCAAGA |
| WRKY33-R | AT2G38470 | GTTTCCCTTCGTAGGTTGTGA |
| WRKY70-01 | AT3G56400 | AACACCAACGCAGAAACTCC |
| WRKY70-02 | AT3G56400 | CGAACCATGATGACGATGAG |
| Control-S | AT2G28390 | aactctatgcagcatttgatccact |
| Control-AS | AT2G28390 | tgattgcatatctttatcgccatc |
