# Supplementary material for: The hnRNP-Q Protein LIF2 Participates in the Plant Immune Response
Source: PLoS One. 2014 Jun 10;9(6):e99343. doi: 10.1371/journal.pone.0099343 (PMC4051675; doi:10.1371/journal.pone.0099343)
Supplement: Table S4 — Parameters for the LC-ESI-MS/MS analysis in negative mode. (DOCX) [file pone.0099343.s006.docx]

**Table S4. Parameters for the LC-ESI-MS/MS analysis in negative mode.**

| Hormone | Retention time (min) | MS/MS transition | Collision Energy (V) | Sampling cone voltage (V) |
| --- | --- | --- | --- | --- |
| D_4_-SA  SA | 12.6 | 141>97  137>93 | 15 | 25 |
| ^13^C_6_-IAA  IAA | 15.3 | 180>136  174>130 | 10 | 16 |
| D_4_-ABA  ABA | 16.6 | 267>156  263>153 | 10 | 25 |
| D_5_-JA  JA | 20.5 | 214>62  209>62 | 15 | 25 |
