# Supplementary material for: The hnRNP-Q Protein LIF2 Participates in the Plant Immune Response
Source: PLoS One. 2014 Jun 10;9(6):e99343. doi: 10.1371/journal.pone.0099343 (PMC4051675; doi:10.1371/journal.pone.0099343)
Supplement: Table S5 — The limit of detection (LOD) and limit of quantification (LOQ). (DOCX) [file pone.0099343.s007.docx]

**Table S5. The limit of detection (LOD) and the limit of quantification (LOQ).**

| Hormone | LOQ (ng/mg FW) | LOD (ng/mg FW) |
| --- | --- | --- |
| ABA | 3.65 | 1.10 |
| AIA | 2.46 | 0.74 |
| JA | 3.07 | 0.92 |
| SA | 2.90 | 0.87 |
